# Adequacy of Empiric Antibiotics Therapy and Its Impact on Outcomes in Adult Critically Ill Sepsis Patients: A Review

**DOI:** 10.21315/mjms2022.29.5.3

**Published:** 2022-10-28

**Authors:** Ahmad Habeeb Hattab Dala Ali Al-Ani, Noordin Othman, Mohamed Azmi Hassali, Baharudin Ibrahim

**Affiliations:** 1 Discipline of Clinical Pharmacy, School of Pharmaceutical Sciences, Universiti Sains Malaysia, Pulau Pinang, Malaysia; 2 College of Pharmacy, Almaarefa University, Riyadh, Saudi Arabia; 3 Department of Clinical and Hospital Pharmacy, College of Pharmacy, Taibah University, Al-Madinah Al-Munawarah, Saudi Arabia; 4 Department of Clinical Pharmacy, Management and Science University, Selangor, Malaysia; 5 Faculty of Pharmacy, Universiti Malaya, Kuala Lumpur, Malaysia

**Keywords:** sepsis, empiric antimicrobial therapy, mortality, predictors, determinants

## Abstract

Sepsis is a medical emergency that involves a systemic immunological response due to an infection, resulting in the end-stage-organs malfunction and death. It is associated with high mortality rate despite a better understanding of the disease pathology and the mechanism involved. This review was designed to summarise the available evidences regarding the adequacy of the empiric antimicrobial therapy (EAMT), its predictors and its impact on the outcomes in intensive care unit (ICU) sepsis patients. Providing an adequate EAMT is considered one of the cornerstones of sepsis management as it has been found to be associated with better survival and is a good predictor for shorter ICU-length-of-stay. In contrast, inadequate EAMT in sepsis patients is associated with poor clinical outcomes including increased mortality and prolonged hospital stay. Evidence from this review suggest that it is important to identify determinants of inadequate EAMT to optimise the antimicrobial therapy provided to sepsis patients. Predictors of inadequate EAMT included co-morbidities (cancer), source and type of infection, higher Acute Physiology and Chronic Health Evaluation (APACHI-II) score and long hospital stay prior to the infection. As EAMT is considered as one of the effective treatment strategies to prevent sepsis associated death, healthcare providers should ensure the adequate antimicrobial therapy is provided for sepsis patients to improve and optimise their management.

## Introduction

Sepsis is a systemic illness that involves the invasion of normally sterile body parts by the microbes (1). It is a medical emergency involving a systemic immunological response due to an infection, resulting in an end stage organ malfunction and death (2). Although the understanding about the sepsis disease process has been established (2), still it is associated with high mortality rate. It has been estimated that sepsis has led to 11 million deaths in 2017 globally. Also, it is the leading cause of death in non-coronary intensive care units (ICUs) in the United States (3, 4).

Antibiotic therapy is considered as one of the effective treatment strategy in the management of sepsis patients (5). Early initiation of the adequate antibiotic therapy has been shown to be associated with lower mortality rates and better survival in bacteremia patients (5–7). This suggests that early initiation of the adequate antibiotic therapy in the moderate to severe infections is beneficial (7). Antibiotic therapy is often guided by empirical evidence particularly in the absence of information regarding causative pathogen or its sensitivity to the antibiotics. Evidently, in severe sepsis and septic shock cases, the initiation of an effective antibiotic therapy within the first hour should be the goal of the therapy (7, 8).

Providing empiric antimicrobial therapy (EAMT) is challenging as it may lead to additional complications (9). Furthermore, inadequate EAMT is considered a life-threatening issue that has often been associated with poor clinical outcomes (9). To understand this aspect more, in this article we reviewed the available literature regarding the adequacy and impact of EAMT on sepsis patients’ outcomes and provide an insight about this critical aspect of sepsis management.

## Methods

To search for studies that described the adequacy of EAMT and its impact on the outcomes of the sepsis patients, we searched PubMed database and google scholar search engine. Terms and their combinations used for searching English-language articles included: adequate, inadequate, appropriate, adequacy, empiric antimicrobial therapy, sepsis, severe sepsis, septic shock, mortality, length of stay, outcomes, survival, ICU and intensive care unit. The inclusion criteria were studies that assessed the adequacy of EAMT and the impact of inadequate EAMT on sepsis ICU patients. We excluded studies which were published in non-English language, assessed impact of EAMT on the outcomes in non-sepsis patients or included non-adult or pediatric sepsis patients.

## Result and Discussion

### Sepsis Associated Mortality

**Figure 1:**
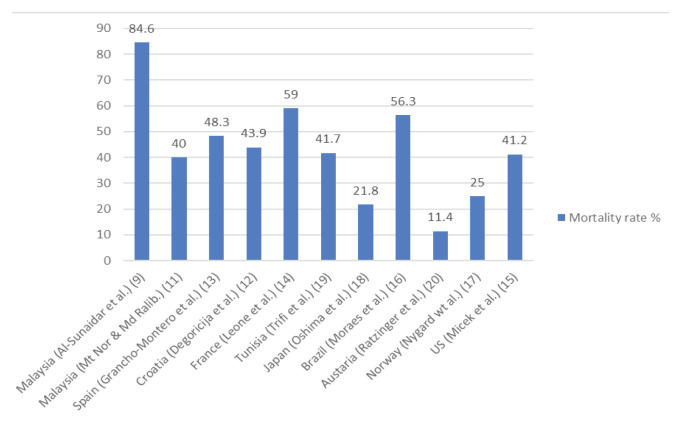
Mortality rates in sepsis patients

Sepsis is considered as a significant public health issue affecting millions of people and represents one of the leading causes of death around the globe (10). Studies that evaluated the mortality rates due to the sepsis in several countries indicated variable results (Figure 1). In Malaysia, sepsis-associated mortality rates were found to be non-consistent. As per the study conducted by Mat Nor and Md Ralib (11), sepsis-associated mortality was 40.0%; on the other hand Al-sunaidar et al. (9) reported sepsis-associated mortality to be almost double (84.6%). Sepsis-associated mortality rates reported from France, Brazil, Spain, Croatia, Tunisia, the United States, Norway and Japan were found to be 59.0%, 56.3%, 48.3%, 43.9%, 41.7%, 41.2%, 25.0% and 21.8%, respectively (12–19). The lowest hospital mortality in sepsis patients was identified in Austria (11.4%) (20). Mortality in sepsis patients can be associated with several causes which are unlikely to be preventable including cancer diseases, heart failure and other co-morbidities (21). Mortality rates in some studies were found to be higher (27.1%–72.1%) in septic shock patients when compared with severe sepsis patients (15.7%–33.7%) and sepsis (17.0%) (12, 18). These findings may partly be explained by the inadequacy of EAMT in the sepsis patients which could be prevented by providing adequate EAMT (21).

### Definition of Empiric Antimicrobial Therapy Adequacy

EAMT refers to the initial antibiotic regimen that is started within 24 h of the admission of the patient (22). There are several studies that have been conducted worldwide to measure the adequacy of the EAMT in the sepsis patients. However, the definition of the EAMT adequacy was inconsistent in these studies (Table 1). For instance, Fitousis et al. (23) have stratified it into appropriateness and accuracy. In this definition, appropriateness referred to an empiric antimicrobial regimen that would cover all suspected organisms based on the suspected site of infection, regardless of the susceptibility data. Accuracy was defined as an empiric antimicrobial regimen that was susceptible to the isolated microorganisms. Most studies defined adequate EAMT as ‘causative microorganism being sensitive to at least one drug administered within 24 h of the culture collection’ or ‘providing an antimicrobial agent(s) in accordance with published guidelines’ or ‘improvement in symptoms’ (Table 1).

### Adequacy of the Empirical Antimicrobial Therapy in Sepsis Patients in the ICU Settings

In the management of the sepsis, therapies are provided to manage the basic elements of the sepsis including infection, organ dysfunction and host response (27). According to the latest surviving sepsis campaign’s international guidelines for the management of sepsis and septic shock, intravenous antimicrobial agents should be started as soon as possible after the recognition of sepsis and it should be within one hour for both sepsis and septic shock (28).

Adequacy of the EAMT has been described by several studies worldwide. Some of these studies have demonstrated that EAMT was provided in an adequate manner in sepsis patients in the ICU settings. These studies showed that adequate EAMT were noted in 90.0%, 82.0%, 81.4% and 89.0% of sepsis patients admitted to the ICU in Canada (25), United States (23), Norway (17) and France (14), respectively. In contrast, lower percentage of adequate EAMT provided to sepsis patients in the ICU setting was seen in many countries. In Tunisia, only 52.0% of the patients were adequately treated with EAMT (19), 27.1% in Malaysia (9) and 58.9% in Austria (20). In addition, we found that there is a lack of studies which described the adequacy of EAMT provided to sepsis patients in the Middle East countries. The percentages of the adequate EAMT provided to sepsis patients reported by the studies around the world have been summarised in Table 2.

### Impact of the Adequacy of Empirical Antimicrobial Therapy in Sepsis Patients on Patient’s Outcomes

Providing an adequate and appropriate EAMT is essential and critical for the treatment of sepsis (29). Providing adequate EAMT to sepsis patients was found to be associated with reduced mortality and increased survival i.e. protective factor (9, 12, 13, 24). However, the impact of the EAMT adequacy on the mortality outcome in sepsis patient seems to be variable in the literature. Several studies have demonstrated a significant association of inadequate EAMT with increased mortality in ICU sepsis patients (9, 19, 20, 26). Moreover, inadequate EAMT was identified as an independent predictor of mortality (15, 17). On the contrary, even though mortality rates were found to be higher in patients who received inadequate EAMT, a significant association between the two was not established in other studies (14, 18, 23).

With regards to the association of EAMT adequacy with the length of hospital stay, most of the studies focused on the mortality outcome. Al-Sunaidar et al. (9) found that providing an appropriate EAMT to critically ill sepsis patients was considered as a good predictor for the decreased ICU-length-of-stay. On the other hand, inadequate EAMT was significantly associated with longer length of stay in both ICU and the hospital (26).

### Determinants of the Adequacy of Empirical Antimicrobial Therapy in Sepsis Patients in the ICU Settings

As discussed in the above sections, several studies have indicated inadequate antimicrobial therapy to be associated with increased mortality in critically ill sepsis patients (9, 19, 20, 26). Therefore, it was important to decipher the risk factors associated with inadequate EAMT. In this context, five studies were found which identified the determinants of the adequacy of the EAMT in sepsis patients (Table 3). Garancho-Montero et al. (5) have found that the presence of fungal infection and previous exposure to the antibiotics as a potential risk factors for inadequate EAMT. This might be due to the fact that the utilization of the antifungal agents is not commonly practiced during admissions to the ICU (5, 30). Also, the previous exposure to the antimicrobials is a risk factor for developing antimicrobial resistance which consequently might lead to the inadequate EAMT (30, 31). According to a matched cohort study, the rate of the nosocomial infection was significantly higher (16.1%) in patients who received inadequate EAMT in comparison to the patients treated adequately with EAMT (3.4%) (26). Besides, cancer patients, poly-microbial infections and higher Acute Physiology and Chronic Health Evaluation (APACHI-II) score were also identified as risk factors of the inadequate EAMT (5, 19, 24, 26, 32).

## Conclusion

Several studies have demonstrated the negative impact of the inadequate EAMT on the outcomes of sepsis patients. According to the retrospective and prospective studies, inadequate EAMT were significantly associated with the poor clinical outcomes in these patients including increased mortality and longer length of hospital stay. As EAMT is considered as one of the preventable causes of sepsis associated death, healthcare providers should ensure that adequate antimicrobial therapy is provided to the patients to improve and optimise the management of sepsis patients. Moreover, hospitals may implement periodically updated empirical antibiotic regimens for the specific sites of infection based on the local microbiology and resistance patterns and according to established practice guidelines to optimise the empirical antimicrobial prescription in sepsis patients.

## Figures and Tables

**Figure 2:**
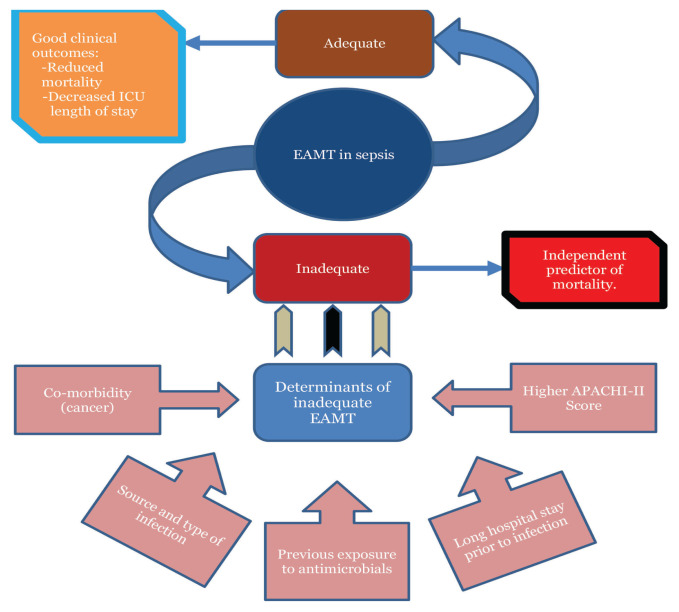
Impact of EAMT adequacy on clinical outcomes of sepsis patients

**Table 1:** Definitions of adequate EAMT used in the literature

Study	According to culture and sensitivity tests	Other
Trifi et al. (19)	√	Improvement in symptoms
Oshima et al. (18)	√	Improvement in symptoms
Moraes et al. (16)	√	According to the local guidelines
Yokota et al. (24)	√	-
Ratzinger et al. (20)	√	-
Nygård et al. (17)	-	According to the local guidelines
Degoricija et al. (12)	√	According to the local guidelines
Micek et al. (15)	√	-
Kanji and Dumaresque (25)	√	According to the local guidelines
Al-Sunaidar et al. (9)	√	According to the local guidelines
Garnacho-Montero et al. (13)	√	According to the local guidelines
Garnacho-Montero et al. (26)	√	According to the local guidelines
Garnacho-Montero et al. (5)	-	According to the local guidelines Two antipseudomonal agents if Pseudomonas aeruginosa was isolated

**Table 2:** Percentage of adequate EAMT provided to sepsis patients

Percentage of adequate EAMT (%)	Country	Reference
52.0	Tunisia	(19)
77.3	Japan	(18)
89.0	Brazil	(16)
58.9	Austria	(20)
81.0	Norway	(17)
82.0	US	(23)
68.7	US	(15)
60.8 (survivors)	Croatia	(12)
30.4 (non-survivors)		
91.0	Canada	(25)
28.1	Malaysia	(9)
69.6 (prior ICU admission)	Spain	(13)
30.4 (after ICU admission)		
91.0	Spain	(26)
83.0	Spain	(5)
89.0	France	(14)

**Table 3:** Factors associated with inadequate EAMT provided to sepsis patients

Factors associated with inadequate EAMT	Reference
Cancer patients	(32)
Nosocomial infections	(26)
Previous exposure to antimicrobials	(5)
Fungal infection	(5)
Poly-microbial infection	(24)
APACHI-II score	(24)
Length of hospital stay prior to infection	(19)
